# A standardised Phase III clinical trial framework to assess therapeutic interventions for Lassa fever

**DOI:** 10.1371/journal.pntd.0010089

**Published:** 2022-01-06

**Authors:** Adebola Tolulope Olayinka, Josephine Bourner, George O. Akpede, Joseph Okoeguale, Chukwuyem Abejegah, Nnennaya A. Ajayi, Christian Akude, Oluwafemi Ayodeji, Daniel G. Bausch, Hilde de Clerck, Chioma Dan-Nwafor, Jake Dunning, Cyril Erameh, Justus Ndulue Eze, Pierre Formenty, Annelies Gillesen, Sulaiman Jalloh, Marie Jaspard, Tolulope Jegede, Jacob Maikere, Denis Malvy, Ephraim Ogbaini-Emovon, Olalekan Ezekial Ojo, Sylvanus Okogbenin, Kwame O’Neill, Maria-Lauretta Orji, Sampson Omagbemi Owhin, Michael Ramharter, Robert J. Samuels, Nathan Shehu, Laura Merson, Alex Paddy Salam, Nzelle Delphine Kayem, Peter Horby, Chikwe Ihekweazu, Piero Olliaro

**Affiliations:** 1 Nigeria Centre for Disease Control, Abuja, Nigeria; 2 Centre for Tropical Medicine and Global Health, Nuffield Department of Medicine, University of Oxford, Oxford, United Kingdom; 3 Irrua Specialist Teaching Hospital, Irrua, Edo, Nigeria; 4 Federal Medical Center, Owo, Ondo State, Nigeria; 5 Alex Ekwueme Federal University Teaching Hospital, Abakaliki, Nigeria; 6 Bingham University Teaching Hospital, Jos, Nigeria; 7 UK Public Health Rapid Support Team, Public Health England, London, United Kingdom; 8 London School of Hygiene & Tropical Medicine, London, United Kingdom; 9 Médecins sans Frontières, Brussels, Belgium; 10 National Infection Service, Public Health England, London, United Kingdom; 11 World Health Organisation, Geneva, Switzerland; 12 Ola During Children’s Hospital, Freetown, Sierra Leone; 13 The Alliance for International Medical Action (ALIMA), Dakar, Senegal; 14 Institut Nationale de la Santé et de la Recherche Medicale (Inserm), Infectious Diseases in Low Income Contries, Unit 1219, Bordeaux, France; 15 Ministry of Health and Sanitation, Freetown, Sierra Leone; 16 Bernhard Nocht Institute for Tropical Medicine, Hamburg, Germany; 17 Department of Medicine, University Medical Center Hamburg-Eppendorf, Hamburg, Germany; 18 Kenema Government Hospital, Kenema, Sierra Leone; 19 Jos University Teaching Hospital, Jos, Plateau State, Nigeria; NIAID Integrated Research Facility, UNITED STATES

## Abstract

**Background:**

Only one recommendation currently exists for the treatment of Lassa fever (LF), which is ribavirin administered in conjunction with supportive care. This recommendation is primarily based on evidence generated from a single clinical trial that was conducted more than 30 years ago–the methodology and results of which have recently come under scrutiny. The requirement for novel therapeutics and reassessment of ribavirin is therefore urgent. However, a significant amount of work now needs to be undertaken to ensure that future trials for LF can be conducted consistently and reliably to facilitate the efficient generation of evidence.

**Methodology:**

We convened a consultation group to establish the position of clinicians and researchers on the core components of future trials. A Core Eligibility Criteria (CEC), Core Case Definition (CCD), Core Outcome Set (COS) and Core Data Variables (CDV) were developed through the process of a multi-stakeholder consultation that took place using a modified-Delphi methodology.

**Results:**

A consensus position was achieved for each aspect of the framework, which accounts for the inclusion of pregnant women and children in future LF clinical trials. The framework consists of 8 core criteria, as well as additional considerations for trial protocols.

**Conclusions:**

This project represents the first step towards delineating the clinical development pathway for new Lassa fever therapeutics, following a period of 40 years without advancement. Future planned projects will bolster the work initiated here to continue the advancement of LF clinical research through a regionally-centred, collaborative methodology, with the aim of delineating a clear pathway through which LF clinical trials can progress efficiently and ensure sustainable investments are made in research capacity at a regional level.

## Author summary

Lassa fever (LF) is an acute viral haemorrhagic fever endemic to West Africa, causing an estimated 500,000 new infections and 10,000 deaths per year. At present, no treatment has been developed to specifically treat LF and only one prospective clinical trial has been conducted to evaluate the current treatment recommendation. Before a new era of LF clinical trials can begin, it is important to develop standardised methods and tools to ensure that trials are conducted in a consistent way and can generate reliable, comparable data. The aim of this project was initiate the first step to improve the comparability of LF studies by developing a standardised Phase III clinical trial methodology for LF therapeutics that could be applied in low-resource settings. Through multi-stakeholder consultation, we established a consensus position of clinicians and researchers on the core components of future Phase III clinical trials: Core Eligibility Criteria (CEC), Core Case Definition (CCD), Core Outcome Set (COS) and Core Data Variables (CDV).

## Introduction

Lassa fever (LF) is an acute viral haemorrhagic illness endemic to West Africa, causing an estimated 500,000 new infections and 10,000 deaths per year. [1] The main animal reservoir of the Lassa virus is the multimammate rat, *Mastomys natalensis*, through which transmission to humans can occur either from direct contact with the rat or indirect contact with contaminated food or household items. [2] Human-to-human transmission is most common in healthcare staff working in resource-limited settings where the supply of high quality protective equipment is suboptimal, preventing consistent adherence to infection prevention and control measures. [3]

The incubation period of LF is wide-ranging, with the onset of symptoms occurring typically between 6 and 21 days. [2] Symptoms are often non-specific and most commonly include fever alongside headache, vomiting and abdominal pain, with a small proportion of patients presenting with clinically severe, life-threatening symptoms, such as seizure, breathing difficulty and shock. [4,5] In hospitalised cases, the case fatality rate (CFR) is reported to be 24–27% [6,7] overall and 34% for pregnant women [8]–however in a recent cohort study taking place in a research setting, the CFR for hospitalised cases was 13% for adults and 6% for children. [9]

Despite a weak evidence base, the primary treatment for LF is ribavirin in conjunction with supportive care. [4,10] This recommendation is largely based on the results of a single prospective clinical trial that was conducted in the 1980s, which has since generated concern about its methods, analysis and the safety of ribavirin when used to treat mild cases of LF. [11–13] The requirement for novel therapeutics, a small number of which have been identified as candidates to be tested, is therefore urgent. [14–16]

However, before a new era of LF clinical trials can begin, robust methods must be developed to ensure that trials are conducted in a consistent way and can generate reliable, comparable data. Given that clinical trial registries show no record of plans to conduct Phase III clinical trials for LF therapeutics in 2021, an opportunity has arisen to set the groundwork for well-conducted trials to begin.

The aim of this project is to improve the comparability of LF studies and accelerate the evaluation of LF therapeutics by outlining the framework–or establishing the foundations–on which future trials can be conducted. We convened a consultation group to establish the position of clinicians and researchers–those who have previous experience of LF patient care or research and who are most likely to be involved in the conduct of future trials–on the core components of LF clinical trials. Specifically, the group were asked to consider the fundamental requirements that a Phase III pivotal trial should incorporate in its eligibility criteria, case definition, outcome measures and data variables to evaluate new and existing therapeutics (**Box 1**).

This project represents the first step towards delineating the clinical development pathway for future LF therapeutics with the aim of guiding wider discussions within the West Africa Lassa fever Consortium (WALC). The WALC plans to build on the work undertaken in this project and expand the scope of this consultation to develop a pre-positioned protocol for Phase II/III clinical trials through consultation with researchers, industry, clinicians, regulators and ethics boards.

**Box 1 –Definitions of the consultation outputs**

The definitions of the four key outputs of the consultation are as follows:

**Core Eligibility Criteria (CEC)**–the characteristics of the study population

**Core Case Definition (CCD)**–how to identify a patient with confirmed Lassa fever

**Core Outcome Set (COS)**–what outcomes to measure in order to assess treatment efficacy

**Core Data Variables (CDV)**–list of recommended data variables for collection in all patients to standardise the characterisation of Lassa fever

The development of COS is supported by the Core Outcome Measures in Effectiveness Trials (COMET) Initiative [17] and the methodology for developing COS is described in the COMET Handbook. [18] The group also recognised the value of developing additional outputs (CEC, CCD and CDV) that would improve the comparability of LF clinical trials.

### Methodology

The project was conducted in two stages. First, a systematic review was carried out to describe the clinical characteristics and outcomes of LF and to guide the consultation process–the results of which have been reported separately. [5] These results generated an initial list of potential inclusion and exclusion criteria, case definitions, patient outcomes and data variables that were included in the first round of the consultation survey. Second, a stakeholder consultation was established to consider the key elements of a clinical trial framework for LF.

We have reported our methodology according to the COS-STAR (Core Outcome Set-STAndards for Reporting) Statement. [19]

### Stakeholder consultation

**Fig 1:**
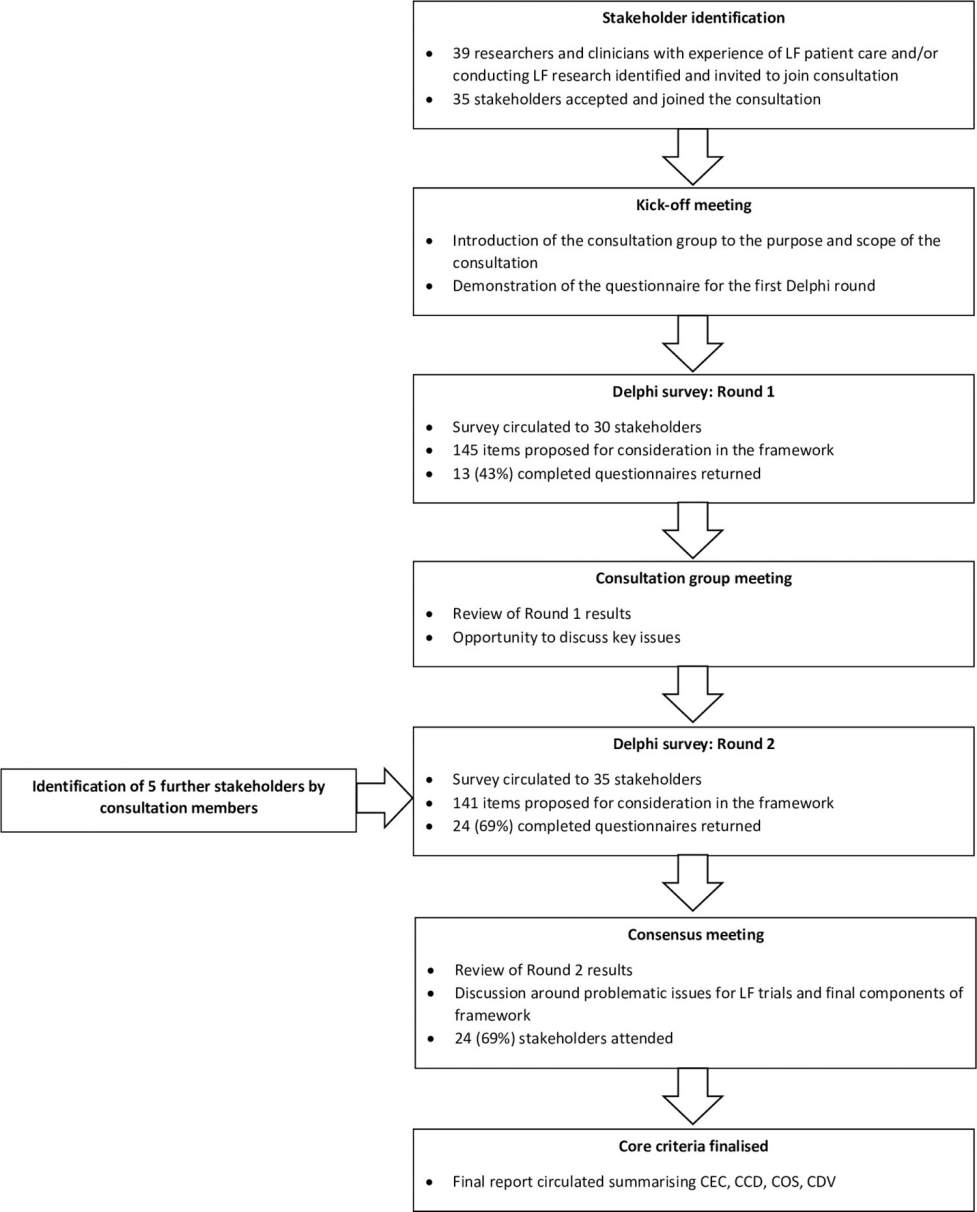
Flowchart illustrating the consultation process.

A stakeholder group was established to collaborate on the development of the core criteria for future trials through a consensus-driven, modified Delphi process (**Fig 1**).

**Fig 2:**
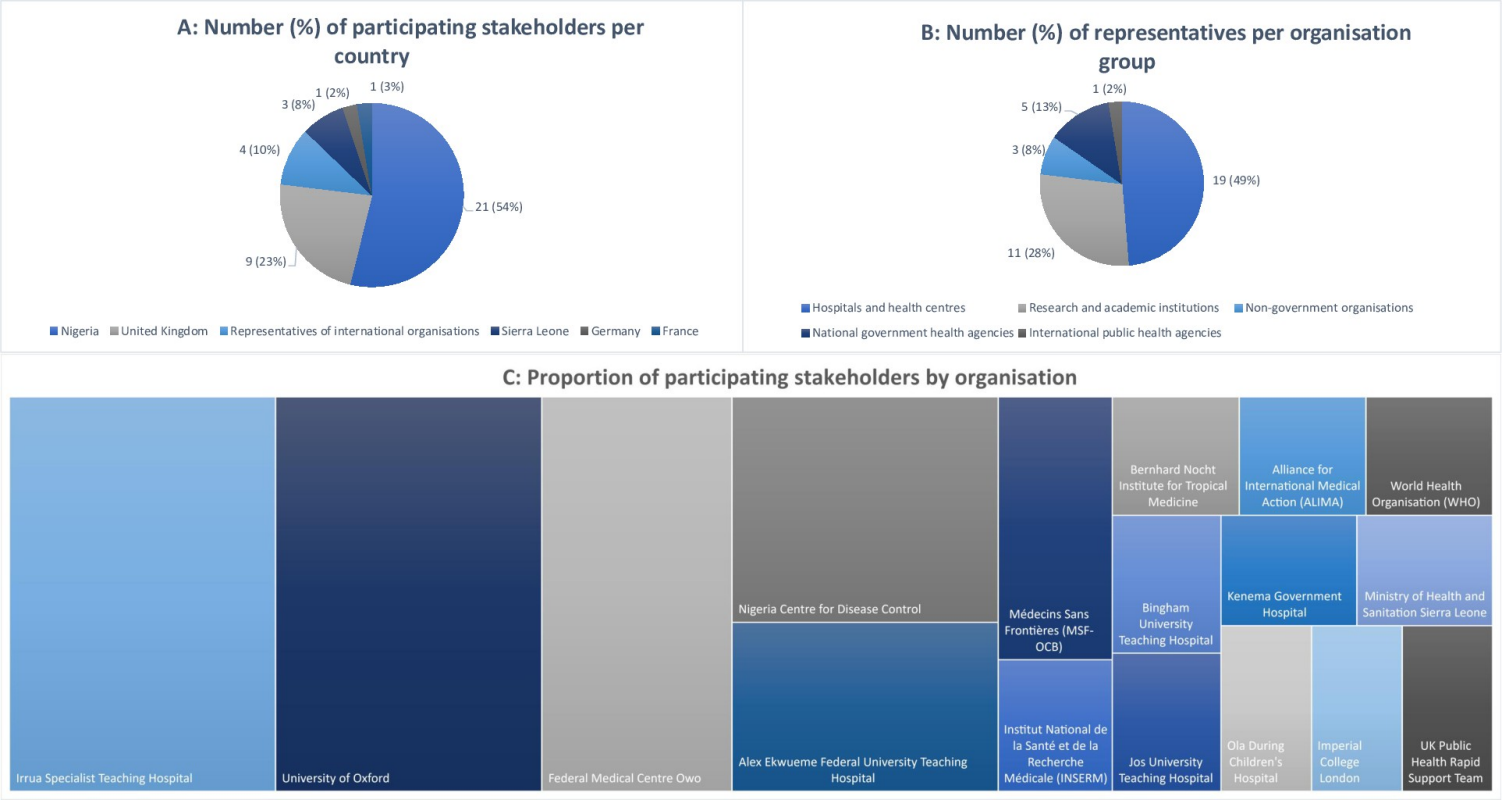
Participating stakeholder summary.

The consultation group consisted of experts in the field of LF, who had experience of LF patient care and/or who had been involved in clinical research for LF. The Nigeria Centre for Disease Control (NCDC) and the International Severe Acute Respiratory and emerging Infection Consortium (ISARIC) assembled the initial list of individuals and organisations that would form the consultation group. This group was expanded following additional proposals from invited members. In total 43 individuals were invited to participate in the consultation group, of whom four declined. The membership of the group covered 16 organisations across 6 countries (**Fig 2**). Of the 39 individuals who agreed to participate, 5 were involved in the central coordination, development and analysis of the consultation surveys and therefore did not complete the questionnaire to avoid bias.

Due to restrictions imposed as a result of the COVID-19 pandemic, it was not possible for the consultation to take place face-to-face as originally planned. The consultation therefore took place remotely over the course of one initial kick-off meeting, two modified Delphi consultations (with two follow-up meetings to discuss results) and one final consensus meeting.

The consultation was conducted using a modified Delphi methodology. The overarching structure of the consultation mirrored a typical Delphi methodology, in that: an expert panel was formed and two rounds of questionnaires were administered with the second questionnaire evolving from the responses to the first questionnaire; and a summary of the responses in each round was anonymously shared with the wider group.

The consultation departed from the traditional structure of a Delphi methodology as a small pilot of the first questionnaire was conducted ahead of the consultation to aid the development of relevant, clear questions and pre-defined responses. Further feedback on the clarity of the first questionnaire was also gathered at the consultation kick-off meeting. Panel meetings were also held between each survey round, in which members could discuss the complex themes that emerged from the survey. A consensus meeting was also held in lieu of a third questionnaire round for panel members to discuss the items that had not gained consensus in the first two rounds and cast final votes on items for inclusion in the framework. In these meetings, it was not possible to ensure the anonymity of participants during discussions; although anonymity was maintained throughout the voting process. The questionnaire used in the second round also used mixed response and scoring types.

### Kick-off meeting

The initial kick-off meeting was organised to explain the structure and scope of the consultation to the participating stakeholders and to demonstrate the questionnaire. Stakeholders were given the opportunity to provide feedback and raise questions about the consultation process. As a result of this meeting, minor modifications were made to the wording and formatting of the questionnaire to ensure clarity.

### Delphi survey

A Delphi-type survey was used to gain consensus on the CEC, CCD, COS and CDV from the consultation group. The questionnaire was developed as an Excel spreadsheet on which stakeholders could respond to the items proposed under each output using a Likert scale, provide additional items for inclusion and provide explanatory comments. An Excel-based questionnaire was chosen over online survey tools to allow participants flexibility to complete the questionnaire over multiple sessions and to maximise participation from members in locations where there weak or unstable internet connections exist.

Consensus was determined when 60% or more of the stakeholders selected the same response. Where 60% or more of the stakeholders stated “Strongly Agree” or “Agree”, items were taken forward to the next Delphi round for further assessment. Where 60% or more of the stakeholders stated “Strongly Disagree” or “Disagree”, items were excluded from the framework. At the end of two Delphi rounds, all remaining items gaining ≥60% agreement for inclusion in the framework were taken forward to a consensus meeting for discussion and a final voting round where inclusion in the framework was determined by the highest vote above 60%.

The first questionnaire contained 145 items–whose inclusion was based on the results of the systematic review–for consideration across all four outputs and was circulated to all stakeholders for completion (**S1; S2**).

Limited decisions were made in the first round to refine the items for inclusion in the framework. To aid the decision-making process, the questionnaire was redeveloped using a mix of scoring systems for the second round and contained 141 items for consideration (**S3**).

All additional items proposed by stakeholders were eligible for inclusion in the next round of the consultation.

Following the analysis of each round, a study report was circulated to all stakeholders and included an anonymised summary of the results (% of stakeholders selecting each score, the group’s mean score and the scoring range) and additional comments provided by the group. These results were also presented to the group at post-survey meetings held between each round during which panel members could discuss issues raised during the survey.

### Consensus meeting

A consensus meeting was held in lieu of a third Delphi-type round, in which the results of the second round were presented and stakeholders discussed and voted on the remaining items to be included in the framework. The final points requiring discussion were as follows: definition of ‘clinical diagnosis of LF’; primary outcome measure and endpoint; measurement instruments for the assessment of ‘Unfavourable Outcome’. Based on feedback from Round 2, workable proposals to navigate the key challenges around these discussion points were developed by the central coordination team as a starting point for discussion.

Following discussion, each proposal was presented using Zoom’s polling function on which stakeholders cast an anonymous vote (**S2**). The poll could be adapted based on discussions and relaunched if amendments were considered necessary. Once all votes were cast, the results were shared (% votes for each option) and stakeholders were given the opportunity to discuss further. The selection gaining the highest number of votes over 60% was included in the framework.

### Analysis

All analyses were performed using Excel. The results of each round were presented using descriptive statistics: N and % of each response per item.

All explanatory comments were presented to stakeholders for further consideration.

## Results

### Round 1

The questionnaire was circulated to 30 stakeholders for completion, 13 of whom returned a completed questionnaire by the deadline, representing a 43% response rate.

Of the 145 items included in this questionnaire, 108 (74%) met the consensus criteria and were taken forward in to the second round of the consultation (**Tables A–D in S1 Data**). 45 new items were proposed for consideration in the next round (**Table E in S1 Data**).

There were no differences in the scoring for pregnant women and children in Round 1, but additional suggestions were provided for these populations in the comments section (**Tables A–D in S1 Data**).

The results of Round 1 and the comments formed the basis of the discussions in the post-survey consultation meeting, during which the group agreed to reformulate the questionnaire and invite further key stakeholders.

### Round 2

In Round 2, 5 stakeholders were added to the group. The questionnaire for Round 2 was therefore circulated to 35 stakeholders in total, 24 (69%) of whom returned a completed questionnaire.

#### Core eligibility criteria

Of the 33 items proposed for the Core Eligibility Criteria in Round 2, 10 items achieved consensus (**Tables G and H in S1 Data**). Across both rounds it was evident that stakeholders did not favour a restrictive list of exclusion criteria, but stated a preference for inclusive eligibility criteria that would not exclude patients on the basis of disease severity or comorbidity or limit recruitment.

Stakeholders agreed that inclusion should be based on a combination of clinical diagnosis and laboratory confirmation. The group also agreed that clinical diagnosis should be based on a list of pre-specified signs and symptoms, of which four (fever, sore throat, hearing loss and bleeding) achieved a consensus from a proposed list of 20. While consensus was achieved according to the analysis plan for these items, the feasibility of identifying eligible patients for a clinical trial based on these four criteria alone was questioned by the analysis team. The earlier systematic review indicated that a low proportion of patients presented with these symptoms (except fever) on admission: sore throat– 44%; hearing loss– 4%; bleeding– 18%. [5] Due to potential low inclusions based on these criteria, the analysis team decided to raise this issue with the stakeholders at the next meeting.

#### Core case definition

Three of the eight proposed items achieved a consensus (**Tables I–K in S1 Data**). All stakeholders agreed that a confirmed case was to be defined on the same basis as the inclusion criteria (clinical diagnosis + laboratory confirmation). All stakeholders also agreed that laboratory confirmation of LF should be conducted using RT-PCR alone and 76% of the stakeholders agreed that sites without access to RT-PCR should not participate in a clinical trial.

#### Core outcome set

Of the 21 proposed outcome measures included in the survey, 4 achieved a consensus for consideration as a primary outcome measure: survival/mortality; progression to severe disease (as defined by presence of either renal failure, encephalopathy, shock, respiratory failure); unfavourable outcome (mortality + progression to severe disease); pregnancy outcome (for pregnant patients only) (**Table L in S1 Data**). In addition, stakeholders were asked to select one preferred primary outcome measure (**Table M in S1 Data**). While no individual primary outcome measure was selected by more than 60% of the stakeholders, “survival/mortality” was selected by 59%. One stakeholder commented that using “survival/mortality” as the primary outcome measure for a clinical trial may not be feasible due to the large sample size required to detect a treatment effect. The primary outcome measure and the feasibility of “survival/mortality” were therefore further debated at the next meeting.

The definitions of four clinical syndromes for inclusion in the characterisation of ‘progression to severe disease’ were also suggested. Two of the proposed syndromes achieved a consensus for their definitions (Acute Kidney Injury and Acute Respiratory Distress Syndrome) (**Tables N and O in S1 Data**). However, revisions were required for encephalopathy and shock due to the lack of an existing standardised definition and feasibility concerns for the proposed measurement instrument, respectively (**Tables P and Q in S1 Data**).

#### Core data variables

Finally, 21 core data variables were identified carried forward in to the consensus meeting (**Table S in S1 Data**).

#### Consensus meeting

Of the 35 stakeholders invited to attend the meeting, 24 participated representing an attendance rate of 69%.

#### Core eligibility criteria

Polling for the definition of ‘clinical diagnosis’ (or ‘clinical suspicion’ in section 1.1) was launched twice as no consensus was achieved in the first poll (**Table U in S1 Data**).

Following discussion and polling, 64% of the stakeholders agreed that clinical suspicion of LF should be based on “history of fever or presence of fever unresponsive to treatment for common illnesses AND at least one of the following: headache/weakness/back pain/ joint pain/ dizziness/sore throat/bleeding/ abdominal pain/ vomiting/diarrhoea/vaginal bleeding/abortion or miscarriage/ unexplained intrauterine death/unexplained breast engorgement) (**Table V in S1 Data**). Members of the group specialising in obstetrics revised the wording relating to pregnancy-specific signs and symptoms following the meeting (**Table 1**).

**Table 1:** Core outputs and definitions included in the final framework.

Item	Criteria	Additional considerations
**Core Eligibility Criteria**		
Clinical suspicion of LF (non-pregnant adults and children)	Non-pregnant adults and children should be enrolled in clinical trials based on clinical suspicion of LF, defined as: • History of fever or presence of fever unresponsive to treatment for common illnesses • AND at least one of the following: headache; weakness; back pain; joint pain; dizziness; sore throat; bleeding; abdominal pain; vomiting; diarrhoea; seizures; haematuria or proteinuria on dipstick urinalysis	At the point of enrolment, a sample should be taken for case confirmation by RT-PCR (see section 1.2) with results returned within 48 hours. Upon receipt of a negative test result for Lassa fever, patients should be immediately removed from the study.
Clinical suspicion of LF (pregnant women)	Pregnant women should be enrolled in clinical trials based on clinical suspicion of Lassa fever, defined as: • History of fever or presence of fever unresponsive to treatment for common illnesses • AND at least one of the following: headache; weakness; back pain; joint pain; dizziness; sore throat; bleeding; abdominal pain; vomiting; diarrhoea; breast swelling or engorgement; unexplained pregnancy loss (miscarriage or intrauterine death); seizures; haematuria or proteinuria on dipstick urinalysis	
Inclusion criteria	Confirmed cases of Lassa fever	See **Core Case Definition**
Exclusion criteria	• Patients receiving end-of-life care for other concomitant conditions • Patients involved in another clinical trial	Additional exclusion criteria will be based on study design and treatments involved.
**Core Case Definition**		
Definition of a confirmed case of LF	Patients who have a positive result on RT-PCR for Lassa fever	Sites that do not have the facilities or capacity to conduct RT-PCR do not qualify for conducting Phase III clinical trials.
**Core Outcome Set**		
Primary Outcome Measure	Unfavourable Outcome: a composite outcome consisting of mortality or deterioration from baseline assessed at Day 14	See **Tables 2 and 3**
Secondary Outcome Measures	• Presence of severe anaemia (defined according to: WHO. Haemoglobin concentrations for the diagnosis of anaemia and assessment of severity. Vitamin and Mineral Nutrition Information System (2011) Geneva, World Health Organization (WHO/NMH/NHD/MNM/11.1)) • Hearing loss • Pregnancy outcome • Time between start of treatment and discharge/recovery • Time between treatment start and delivery/miscarriage (only for trials enrolling pregnant patients) • Time between delivery/miscarriage and hospital discharge/recovery (only for trials enrolling pregnant patients)	
**Core Data Variables**		
Signs, symptoms and assessments critical to evaluate treatment safety and/or efficacy	• Date of symptom onset • Date of contact with a confirmed case (optional) • Frank bleeding • Pregnancy complications (including vaginal bleeding in pregnancy and excessive bleeding in labour) • Level of consciousness (ACVPU or GCS, as specified in **Tables 2** and **3**) • Seizure • Temperature • Blood pressure • Creatinine • Urine output (if creatinine testing is not available) • Partial pressure of oxygen (PaO_2_) • Fraction of inspired oxygen (FiO_2_) • Oxygen saturation (SpO_2_) • Blood urea nitrogen • Aspartate aminotransferase • Alanine transaminase • Pulse • Potassium • Haemoglobin • Point of care ultrasound (pregnant patients only)	Data on other signs and symptoms (that meet the CTCAE definition for Grade 3 or Grade 4) or assessments that were conducted from the point of inclusion until patient outcome should also be collected.

#### Core outcome set

Sample size simulations were shown to the stakeholders to guide discussions about the feasibility of including survival/mortality as the primary outcome measure. It was widely agreed that the sample sizes presented would not be achievable for LF and, upon voting, the majority of stakeholders (83%) agreed that ‘unfavourable outcome’–a composite primary outcome measure–is a more feasible primary outcome measure (**Table W in S1 Data**). 84% of stakeholders agreed that the assessment of the primary outcome measure should take place at 14 days post-enrolment (**Table X in S1 Data**).

Measurement instruments for the four pathologies characterising ‘unfavourable outcome’ (Acute Kidney Injury, Acute Respiratory Distress Syndrome, shock, and encephalopathy) were proposed for evaluation.

64% of the group agreed that Acute Kidney Injury (AKI) should be assessed on the basis of either the Sequential Organ Failure Assessment (SOFA) score [20] or urine output alone (where creatinine testing is not possible) (**Table Y in S1 Data**).

68% of the stakeholders agreed that Acute Respiratory Distress Syndrome (ARDS) should be assessed by SOFA score using one of two methods (partial pressure of oxygen (PaO2)/fraction of inspired oxygen (FiO2) or oxygen saturation (SpO2)/FiO2)) (**Table Z in S1 Data**).

The majority of stakeholders (76%) agreed that shock can be assessed by SOFA score (with the option to not record inotropes if unavailable) (**Table AA in S1 Data**).

Following discussions and multiple rounds of voting, stakeholders agreed that encephalopathy should be assessed on the basis of ‘ACVPU + seizures’ (**Table AB in S1 Data**).

The definition of ‘unfavourable outcome’ for each syndrome will be delineated in future work.

The group also agreed that an adapted assessment system for paediatric patients was required.

The final CEC, CCD, COS, CDV are presented in **Tables 1–3**.

**Table 2:** Definition of Unfavourable Outcome (adults).

Body system	Renal	Respiratory	Cardiovascular	Nervous
Pathology	AKI (Acute kidney injury)	ARDS (Acute respiratory distress syndrome)	Shock	Encephalopathy
Assessment method	Creatinine, Urine output	Arterial blood gas (ABG) analysis: PaO2 or Pulse oximetry: SpO2	Blood Pressure: Mean Arterial Pressure (MAP)	ACVPU [21]
Acceptable definitions	SOFA [22] 0–4 (creatinine test preferred; where creatinine testing is not available, urine output is acceptable provided it is measured accurately)	SOFA [22] 0–4 1. If ABG available: PaO2/FiO2 2. if ABG not available: SpO2/FiO2	1. SOFA [22] 0–4 (if inotropes used, dose recorded; if not available tick box ‘not available’)	ACVPU [21] + seizure

**Table 3:** Definition of Unfavourable Outcome (children).

Body system	Renal	Respiratory	Cardiovascular	Nervous
Pathology	AKI (Acute kidney injury)	ARDS (Acute respiratory distress syndrome)	Shock	Encephalopathy
Assessment method	Creatinine, Urine output	Arterial blood gas (ABG) analysis: PaO2 or Pulse oximetry: SpO2	Blood Pressure: Mean Arterial Pressure (MAP)	Paediatric GCS [23]
Acceptable definitions	pSOFA [22] 0–4 (creatinine test preferred; where creatinine testing is not available, urine output is acceptable provided it is measured accurately)	pSOFA [22] 0–4 1. If ABG available: PaO2/FiO2 2. if ABG not available: SpO2/FiO2	1. pSOFA [22] 0–4 (if inotropes used, dose recorded; if not available tick box ‘not available’)	Paediatric GCS [23] +/- seizure (single prolonged >15 min, or multiple)

## Discussion

This project represents the first step towards delineating the clinical development pathway for new Lassa fever therapeutics, following a period of 40 years without advancement.

We have presented the consensus position of clinicians and researchers, from a range of health agencies, humanitarian organisations and academic institutions, on the core criteria for future pivotal clinical trials for LF. The multi-stakeholder approach is critical to prepare the groundwork for the collaborative and coordinated progression of LF trials and ensure the buy-in of potential investigators and treatment centres. Establishing these partnerships is crucial to avoid a piecemeal approach to conducting trials for a disease that requires an efficient coordinated research strategy. Prioritising an efficient clinical trial pathway will also optimise the use of the limited resources that are available to conduct studies of this nature.

The breadth of this framework also highlights the need to ensure key populations–specifically, pregnant women and children, who are disproportionately affected by LF and face increased risks–are included in the clinical development pathway for new therapeutics. [8,24]

### Limitations

The scope of this project was limited to collating the perspectives of clinicians and researchers. To increase the utility and specificity of the core criteria outlined above, obtaining input from regulators and ethics boards will be critical in future work. In particular, identifying suitable primary outcome measures for pivotal clinical trials for diseases, such as Lassa fever–that have low, sporadic case notification and low mortality, and which may be hampered by suboptimal surveillance systems–is challenging and will benefit from collaboration with these groups. The use of hard endpoints–those that are typically used in pivotal trials like mortality–are not feasible for LF due to the large sample size required. Therefore, discussions will need to be initiated about the acceptability of composite and surrogate endpoints.

Equally, due to restrictions enforced as a result of the COVID-19 pandemic, it was not possible to involve survivors of LF in the development of the framework at this stage. Funding has been secured to complete this activity and collect data on patients’ expectations of LF clinical trials and therapeutics towards the end of 2021.

### Future work

The West Africa Lassa fever Consortium (WALC) will progress the work described here through a multi-stakeholder, platform approach that aims to build capacity in regional research centres and support the sustainable uptake of future clinical trials by local research groups.

The stakeholders and scope of the consortium have been expanded beyond this project to include national and international regulators, ethics boards, health agencies and social scientists who, alongside clinicians and researchers, will generate a clinical development plan and a pre-positioned protocol for future pivotal Phase II/III LF trials, among other outputs. The core criteria developed within the scope of this project will therefore be reviewed by the consortium to obtain the necessary specificity for implementation in pharmaceutical trials aiming to establish the safety and efficacy of new drugs.

Social science studies have been incorporated in to the WALC’s workplan to enhance the acceptability and uptake of the consortium’s outputs among both patients and clinicians. The project will engage survivors of LF on topics such as their patient journey to enhance the eligibility criteria and inform the development of clinically relevant primary and secondary outcome measures.

Planned future work will also involve working directly with regulatory authorities to identify a clear regulatory pathway for the inclusion of pregnant women and children in clinical trials for novel therapeutics.

Overall the WALC aims to bolster the work initiated in this project to continue the advancement of LF clinical research through a regionally-centred, collaborative methodology and to delineate a clear pathway through which LF clinical trials can progress efficiently ensuring sustainable investments are made in research capacity at a regional level.

## Supporting information

S1 Table Delphi survey: Round 1. (XLSX)

S2 Table Extended methodology. (DOCX)

S3 Table Delphi survey: Round 2. (XLSX)

S1 Data Consultation results. (DOCX)
