# Supplementary material for: A standardised Phase III clinical trial framework to assess therapeutic interventions for Lassa fever
Source: PLoS Negl Trop Dis. 2022 Jan 6;16(1):e0010089. doi: 10.1371/journal.pntd.0010089 (PMC8769305; doi:10.1371/journal.pntd.0010089)
Supplement: S2 Table — (DOCX) [file pntd.0010089.s002.docx]

**Supplementary material - extended methodology**

***Round 1***

Stakeholders were asked to consider the inclusion of 145 items proposed for the CEC, CCD, COS and CDV and provide responses on a 5-point Likert scale (“strongly agree” to “strongly disagree”). Stakeholders were asked to provide responses for three key LF populations: a ‘general’ trial population (non-pregnant adult), paediatric population and for pregnant women. A response to all items in the survey was required for the ‘general’ population. However, stakeholders were instructed to enter a response for pregnant women and children if their responses differed from that of a general trial population.

***Round 2***

A 5-point Likert scale was used to include top-level items in the framework (i.e. “Clinical diagnosis is defined as the presence of fever that has not responded to treatment, plus ONE OR MORE of the pre-specified signs and symptoms”). Where definitions of top-level items were required (i.e. to develop the aforementioned pre-specified list of signs and symptoms), an alternative scoring system was implemented (i.e. scoring options for pre-specified signs and symptoms were ‘fundamental to the diagnosis of LF’ or ‘not usually indicative of LF in the absence of other symptoms’). Alternative scoring systems were developed for other items to ensure clarity in decision making and to under the feasibility of proposals.

As there were no differences in the responses between the general, paediatric and pregnant women populations in Round 1, stakeholders were requested to complete the survey for a general population only in Round 2 and indicate in the comments whether their given response would change for pregnant women and/or children.

***Consensus meeting***

Following analysis of the results in Round 2, the coordinators of the project felt that discussion was required to finalise the remaining items in the framework, so a consensus meeting was held in place of a third Delphi-type survey. The meeting took place via Zoom, using the polling function to collect anonymous votes on a pre-set questionnaire (**Box S1**) developed following the analysis of the Round 2 survey.

**Box S1 – Items for consideration and voting options for the consensus meeting**

**Item 1**: Definition of ‘clinical diagnosis’

**Question**: How should ‘clinical diagnosis’ of Lassa fever be defined in a Phase III clinical trial?

**Voting Options**:

1. no restrictions on signs and symptoms in order for clinical diagnosis to be made
2. presence of fever AND [(at least one of the following: headache/back pain/joint pain) OR (at least one digestive symptom: abdominal pain, vomiting, diarrhoea)]
3. presence of fever AND [at least one of the following: headache/weakness/back pain/ joint pain/ dizziness/sore throat/bleeding) OR (at least one digestive symptom: abdominal pain, vomiting, diarrhoea)]
4. presence of fever AND at least one of the following: sore throat/bleeding from orifices/ hearing loss
5. in the absence of fever, presence of [(at least one of the following: headache/backache/joint pain) AND (at least one digestive symptom: abdominal pain, vomiting, diarrhoea)]

**Item 2**: Considering the sample size required for a clinical trial where survival/mortality is the Primary Outcome Measure, do you agree that ‘Unfavourable Outcome’ is a more feasible Primary Outcome Measure?

**Voting Options**:

1. Yes
2. No

**Item 3**: Is 14 days an appropriate timepoint to assess the Primary Outcome Measure?

**Voting Options**:

1. Yes
2. No

**Item 4**: Which proposal do you prefer to assess Acute Kidney Injury?

**Voting Options**

1. SOFA 0-4 (creatinine or urine output)
2. SOFA 0-4 OR urine output alone are acceptable
3. Urine output alone

**Item 5**: Which proposal do you prefer to assess Acute Respiratory Distress Syndrome?

**Voting Options**:

1. SOFA 0-4 (PaO_2_/FiO_2_)
2. SOFA 0-4 (PaO_2_/FiO_2_) OR SOFA 0-4 (SF [SPO_2_/FIO_2_]) are acceptable
3. SOFA 0-4 (SF [SPO_2_/FIO_2_])

**Item 6**: Which proposal do you prefer to assess shock?

Voting Options:

1. SOFA 0-4
2. SOFA 0-4 OR SOFA 0-4 (with option to not record inotropes if unavailable) are acceptable
3. SOFA 0-4 (with option to not record inotropes if unavailable)

**Item 7**: Which proposal do you prefer to assess encephalopathy?

Voting Options:

1. SOFA 0-4 (Glasgow Coma Scale)
2. SOFA 0-4 (Glasgow Coma Scale) OR AVPU score
3. AVPU score

Template polling questions and options were also developed in advance to anticipate further polling as a result of discussions that took place during the meeting (**Box S2**). These questions could be launch at any point during the meeting to capture responses to ad-hoc verbal questions.

B**ox S2 – Template questions and answers prepared in advance of the meeting**

1. **Do you agree?**
2. Yes
3. No
4. **Select one option:**
5. Option 1
6. Option 2
7. Option 3

During discussions about the CEC, the definition of ‘clinical diagnosis’ was manually adapted on the slide set as stakeholders agreed that Item 1 (**described in Box S1**) required further clarification. Options 2 and 3 were expanded to ensure they incorporated clinical suspicion in pregnant women who may exhibit symptoms unique to LF in pregnancy on admission.

**Box S3 – modified voting options for the definition of ‘clinical diagnosis’ following stakeholder feedback**

Voting Options*:

1. no restrictions on signs and symptoms in order for clinical diagnosis to be made
2. history of fever or presence of fever unresponsive to treatment for common illnesses AND [at least one of the following: headache/weakness/back pain/ joint pain/ dizziness/sore throat/bleeding) OR (at least one digestive symptom: abdominal pain, vomiting, diarrhoea)] – expanded to include signs and symptoms associated with Lassa fever in pregnancy (vaginal bleeding; abortion or miscarriage; unexplained intrauterine death; unexplained breast engorgement)
3. history of fever or presence of fever unresponsive to treatment for common illnesses, presence of [(at least one of the following: headache/weakness/back pain/ joint pain/ dizziness/sore throat/bleeding) AND (at least one digestive symptom: abdominal pain, vomiting, diarrhoea)] - expanded to include signs and symptoms associated with Lassa fever in pregnancy (vaginal bleeding; abortion or miscarriage; unexplained intrauterine death; unexplained breast engorgement)

*Note: modified elements are denoted by red text
