# Supplementary material for: A standardised Phase III clinical trial framework to assess therapeutic interventions for Lassa fever
Source: PLoS Negl Trop Dis. 2022 Jan 6;16(1):e0010089. doi: 10.1371/journal.pntd.0010089 (PMC8769305; doi:10.1371/journal.pntd.0010089)
Supplement: S1 Data — (DOCX) [file pntd.0010089.s004.docx]

**Supplementary material – Consultation results**

***Results of Round 1***

**Table A – Frequency and mean of responses to items proposed for inclusion in the Core Eligibility Criteria**

| CORE ELIGIBILITY CRITERIA | N (%) | | | | |
| --- | --- | --- | --- | --- | --- |
|  | Strongly agree | Agree | No preference | Disagree | Strongly disagree |
| Virological confirmation | 4 (36) | 5 (45) | 2 (18) | 0 (0) | 0 (0) |
| Virological confirmation and symptoms consistent with Lassa Fever | 9 (75) | 1 (8) | 2 (17) | 0 (0) | 0 (0) |
| Presence of fever which has not responded to treatment in >48 hours and one or more of the following signs or symptoms: | 5 (42) | 2 (17) | 2 (17) | 3 (25) | 0 (0) |
| 3a) Back or joint pain | 2 (25) | 3 (38) | 2 (25) | 1 (13) | 0 (0) |
| 3b) Weakness | 5 (63) | 2 (25) | 0 (0) | 1 (13) | 0 (0) |
| 3c) Headache | 1 (13) | 4 (50) | 2 (25) | 1 (13) | 0 (0) |
| 3d) Chest pain | 0 (0) | 5 (63) | 2 (25) | 1 (13) | 0 (0) |
| 3e) Dizziness | 0 (0) | 2 (25) | 6 (75) | 0 (0) | 0 (0) |
| 3f) Malaise | 3 (38) | 3 (38) | 2 (25) | 0 (0) | 0 (0) |
| 3g) Sore throat | 5 (63) | 3 (38) | 0 (0) | 0 (0) | 0 (0) |
| 3h) Abdominal pain | 3 (38) | 3 (38) | 0 (0) | 2 (25) | 0 (0) |
| 3i) Vomiting | 2 (22) | 4 (44) | 1 (11) | 2 (22) | 0 (0) |
| 3j) Nausea | 3 (33) | 2 (22) | 3 (33) | 1 (11) | 0 (0) |
| 3k) Diarrhoea | 1 (11) | 5 (56) | 2 (22) | 1 (11) | 0 (0) |
| 3l) Bleeding from orifices | 6 (67) | 3 (33) | 0 (0) | 0 (0) | 0 (0) |
| 3m) Generalised muscle pain | 1 (11) | 8 (89) | 0 (0) | 0 (0) | 0 (0) |
| Onset of symptom(s) within specified timeframe before presentation | 4 (33) | 3 (25) | 3 (25) | 2 (17) | 0 (0) |
| 5a) Symptom onset within 7 days prior to presentation | 3 (38) | 4 (50) | 1 (13) | 0 (0) | 0 (0) |
| 5b) Symptom onset within 14 days prior to presentation | 2 (22) | 6 (67) | 1 (11) | 0 (0) | 0 (0) |
| No specified timeframe from onset to presentation | 2 (18) | 3 (27) | 1 (9) | 5 (45) | 0 (0) |
| Exclusion of patients with coinfection | 1 (8) | 2 (17) | 2 (17) | 6 (50) | 1 (8) |
| No generalisable exclusion criteria | 1 (8) | 6 (50) | 1 (8) | 3 (25) | 1 (8) |
| Exclusion criteria should only be dependent on study design and treatments involved | 2 (17) | 6 (50) | 0 (0) | 4 (33) | 0 (0) |

**Table B – Frequency and mean of responses to items proposed for inclusion in the Core Case Definition**

| CORE CASE DEFINITION | N (%) | | | | |
| --- | --- | --- | --- | --- | --- |
|  | Strongly agree | Agree | No preference | Disagree | Strongly disagree |
| Laboratory confirmation alone | 1 (8) | 2 (17) | 2 (17) | 7 (58) | 0 (0) |
| Laboratory confirmation and clinical diagnosis | 11 (85) | 1 (8) | 0 (0) | 1 (8) | 0 (0) |
| Clinical diagnosis alone only where the setting does not permit laboratory confirmation | 1 (8) | 5 (42) | 2 (17) | 2 (17) | 2 (17) |
| RT-PCR | 12 (92) | 1 (8) | 0 (0) | 0 (0) | 0 (0) |
| Viral culture | 1 (8) | 5 (42) | 4 (33) | 2 (17) | 0 (0) |
| IFA | 0 (0) | 2 (17) | 5 (42) | 4 (33) | 1 (8) |
| ELISA IgM | 0 (0) | 7 (58) | 1 (8) | 4 (33) | 0 (0) |
| ELISA Ag | 0 (0) | 3 (25) | 6 (50) | 3 (25) | 0 (0) |
| ELISA IgG | 0 (0) | 2 (17) | 2 (17) | 6 (50) | 2 (17) |
| CF | 0 (0) | 2 (18) | 5 (45) | 2 (18) | 2 (18) |
| IHC | 0 (0) | 1 (9) | 6 (55) | 2 (18) | 2 (18) |

**Table C – Frequency and mean of responses to items proposed for inclusion in the Core Outcome Set**

| CORE OUTCOME SET | N (%) | | | | |
| --- | --- | --- | --- | --- | --- |
|  | Strongly agree | Agree | No preference | Disagree | Strongly disagree |
| Survival/ mortality | 9 (75) | 3 (25) | 0 (0) | 0 (0) | 0 (0) |
| 14 days | 4 (50) | 3 (38) | 1 (13) | 0 (0) | 0 (0) |
| 21 days | 1 (20) | 3 (60) | 1 (20) | 0 (0) | 0 (0) |
| 28 days | 2 (29) | 3 (43) | 1 (14) | 1 (14) | 0 (0) |
| Progression to severe disease | 3 (30) | 6 (60) | 0 (0) | 0 (0) | 1 (10) |
| 28a) Renal failure | 8 (67) | 4 (33) | 0 (0) | 0 (0) | 0 (0) |
| 28b) Encephalopathy | 8 (67) | 4 (33) | 0 (0) | 0 (0) | 0 (0) |
| 28c) Shock | 5 (42) | 6 (50) | 1 (8) | 0 (0) | 0 (0) |
| 28d) Respiratory failure | 5 (42) | 5 (42) | 1 (8) | 1 (8) | 0 (0) |
| Progression to severe disease based on clinical assessment of unspecified criteria | 1 (8) | 3 (23) | 5 (38) | 4 (31) | 0 (0) |
| "Unfavourable outcome" (composite outcome of mortality AND progression to severe disease | 4 (31) | 7 (54) | 2 (15) | 0 (0) | 0 (0) |
| Serious Adverse Events (SAEs) (i.e. events that result in hospitalisation, death, incapacity or disability, congenital abnormality/ birth defect, are considered life-threatening or otherwise medically significant) | 4 (31) | 5 (38) | 3 (23) | 1 (8) | 0 (0) |
| Grade 3 and 4 adverse events (grade 3 or 4 adverse events that do not meet the definition of 'serious' as described in item 28) | 0 (0) | 5 (42) | 6 (50) | 1 (8) | 0 (0) |
| No secondary outcome | 0 (0) | 0 (0) | 0 (0) | 7 (64) | 4 (36) |

**Table D – Frequency and mean of responses to items proposed for inclusion in the Core Data Variables**

| CORE DATA VARIABLES | N (%) | | | | |
| --- | --- | --- | --- | --- | --- |
|  | Strongly agree | Agree | No preference | Disagree | Strongly disagree |
| Date of symptom onset | 7 (70) | 3 (30) | 0 (0) | 0 (0) | 0 (0) |
| Date of contact with confirmed case (if applicable) | 4 (40) | 5 (50) | 1 (10) | 0 (0) | 0 (0) |
| Date of travel to an endemic area (if applicable) | 3 (30) | 5 (50) | 1 (10) | 1 (10) | 0 (0) |
| Signs and symptoms assessed at baseline | 6 (60) | 4 (40) | 0 (0) | 0 (0) | 0 (0) |
| 38a) Fever | 10 (83) | 2 (17) | 0 (0) | 0 (0) | 0 (0) |
| 38b) Weakness | 6 (50) | 6 (50) | 0 (0) | 0 (0) | 0 (0) |
| 38c) Dizziness | 2 (17) | 8 (67) | 1 (8) | 1 (8) | 0 (0) |
| 38d) Muscle pain | 3 (25) | 9 (75) | 0 (0) | 0 (0) | 0 (0) |
| 38e) Chest pain | 2 (17) | 5 (42) | 4 (33) | 1 (8) | 0 (0) |
| 38f) Back pain | 3 (25) | 5 (42) | 4 (33) | 0 (0) | 0 (0) |
| 38g) Joint pain | 1 (8) | 9 (75) | 2 (17) | 0 (0) | 0 (0) |
| 38h) Malaise | 4 (22) | 7 (58) | 1 (8) | 0 (0) | 0 (0) |
| 38i) Sore throat | 8 (67) | 4 (33) | 0 (0) | 0 (0) | 0 (0) |
| 38j) Abdominal pain | 4 (33) | 7 (58) | 1 (8) | 0 (0) | 0 (0) |
| 38k) Vomiting | 2 (17) | 8 (67) | 1 (8) | 1 (8) | 0 (0) |
| 38l) Nausea | 3 (25) | 6 (50) | 2 (17) | 1 (8) | 0 (0) |
| 38m) Diarrhoea | 1 (8) | 9 (75) | 2 (17) | 0 (0) | 0 (0) |
| 38n) Lymphadenopathy | 0 (0) | 4 (33) | 6 (50) | 2 (17) | 0 (0) |
| 38o) Location of adenopathy | 1 (9) | 2 (18) | 6 (55) | 2 (18) | 0 (0) |
| 38p) Bleeding | 9 (75) | 3 (25) | 0 (0) | 0 (0) | 0 (0) |
| 38q) Location of bleeding | 5 (42) | 6 (50) | 1 (8) | 0 (0) | 0 (0) |
| 38r) Level of consciousness (alert, confusion, voice, pain, unresponsive) | 6 (50) | 4 (33) | 2 (17) | 0 (0) | 0 (0) |
| 38s) Seizure | 4 (33) | 5 (42) | 3 (25) | 0 (0) | 0 (0) |
| 38t) Breathing difficulty | 2 (17) | 6 (50) | 4 (33) | 0 (0) | 0 (0) |
| 38u) Shock | 3 (25) | 8 (67) | 1 (8) | 0 (0) | 0 (0) |
| 38v) Renal failure | 7 (58) | 5 (42) | 0 (0) | 0 (0) | 0 (0) |
| 38w) Encephalopathy | 6 (50) | 5 (42) | 1 (8) | 0 (0) | 0 (0) |
| 38x) Respiratory failure | 3 (25) | 5 (42) | 4 (33) | 0 (0) | 0 (0) |
| The number of days for which each sign/symptom has been present | 5 (42) | 6 (50) | 1 (8) | 0 (0) | 0 (0) |
| To indicate presence, all signs and symptoms should be graded | 1 (9) | 3 (27) | 4 (36) | 3 (27) | 0 (0) |
| To indicate presence, signs and symptoms should be assessed as 'present' or 'not present' | 1 (8) | 10 (83) | 0 (0) | 0 (0) | 1 (8) |
| Assessment of vital signs at baseline | 10 (83) | 2 (17) | 0 (0) | 0 (0) | 0 (0) |
| 42a) Respiratory rate | 6 (50) | 6 (50) | 0 (0) | 0 (0) | 0 (0) |
| 42b) Pulse | 8 (67) | 4 (33) | 0 (0) | 0 (0) | 0 (0) |
| 42c) Blood pressure | 8 (67) | 4 (33) | 0 (0) | 0 (0) | 0 (0) |
| 42d) Temperature | 8 (67) | 4 (33) | 0 (0) | 0 (0) | 0 (0) |
| 42e) Oxygen saturation | 6 (50) | 4 (33) | 2 (17) | 0 (0) | 0 (0) |
| 42f) Cardiac function (ECG) | 1 (8) | 5 (42) | 6 (50) | 0 (0) | 0 (0) |
| 42g) AST | 7 (58) | 5 (42) | 0 (0) | 0 (0) | 0 (0) |
| 42h) ALT | 7 (58) | 4 (33) | 1 (8) | 0 (0) | 0 (0) |
| 42i) Creatinine | 10 (83) | 2 (17) | 0 (0) | 0 (0) | 0 (0) |
| 42j) BUN | 8 (67) | 4 (33) | 0 (0) | 0 (0) | 0 (0) |
| 42k) Urine protein | 6 (50) | 5 (42) | 1 (8) | 0 (0) | 0 (0) |
| Laboratory diagnotics should be performed via any method available to the research team | 1 (8) | 1 (8) | 3 (25) | 6 (50) | 1 |
| Laboratory diagnotics should be limited to certain methods | 3 (25) | 7 (58) | 2 (17) | 0 (0) | 0 (0) |
| 44a) RT-PCR | 8 (80) | 2 (20) | 0 (0) | 0 (0) | 0 (0) |
| 44b) Viral culture | 0 (0) | 6 (60) | 2 (20) | 2 (20) | 0 (0) |
| 44c) IFA | 0 (0) | 3 (30) | 3 (30) | 4 (40) | 0 (0) |
| 44d) IgM ELISA | 0 (0) | 7 (70) | 1 (10) | 2 (20) | 0 (0) |
| 44e) Ag ELISA | 0 (0) | 3 (30) | 5 (50) | 2 (20) | 0 (0) |
| 44f) IgG ELISA | 0 (0) | 4 (40) | 2 (20) | 3 (30) | 1 (10) |
| 44g) CF | 0 (0) | 2 (25) | 3 (38) | 3 (38) | 0 (0) |
| 44h) IHC | 0 (0) | 1 (13) | 4 (50) | 3 (38) | 0 (0) |
| Single testing methods are appropriate | 0 (0) | 6 (60) | 4 (40) | 0 (0) | 0 (0) |
| Combined testing methods should be used where possible | 3 (27) | 5 (45) | 2 (18) | 1 (9) | 0 (0) |
| Testing for malaria should always be carried out when trials are being conducted in a malaria-endemic area | 9 (82) | 2 (18) | 0 (0) | 0 (0) | 0 (0) |
| Testing for HIV should be carried out in all settings | 1 (9) | 4 (36) | 5 (45) | 1 (9) | 0 (0) |
| Signs and symptoms assessed at post-baseline timepoints: | 5 (50) | 5 (50) | 0 (0) | 0 (0) | 0 (0) |
| 49a) Fever | 6 (55) | 5 (45) | 0 (0) | 0 (0) | 0 (0) |
| 49b) Weakness | 3 (27) | 7 (64) | 0 (0) | 1 (9) | 0 (0) |
| 49c) Dizziness | 2 (18) | 7 (64) | 1 (9) | 1 (9) | 0 (0) |
| 49d) Muscle pain | 2 (18) | 7 (64) | 1 (9) | 1 (9) | 0 (0) |
| 49e) Chest pain | 1 (9) | 5 (45) | 3 (27) | 2 (18) | 0 (0) |
| 49f) Back pain | 1 (9) | 6 (64) | 3 (27) | 1 (9) | 0 (0) |
| 49g) Joint pain | 1 (9) | 7 (64) | 2 (18) | 1 (9) | 0 (0) |
| 49h) Malaise | 4 (36) | 6 (55) | 0 (0) | 1 (9) | 0 (0) |
| 49i) Sore throat | 3 (27) | 7 (64) | 0 (0) | 1 (9) | 0 (0) |
| 49j) Abdominal pain | 1 (9) | 9 (82) | 0 (0) | 1 (9) | 0 (0) |
| 49k) Vomiting | 3 (27) | 6 (55) | 0 (0) | 2 (18) | 0 (0) |
| 49l) Nausea | 1 (9) | 6 (55) | 2 (18) | 2 (18) | 0 (0) |
| 49m) Diarrhoea | 2 (18) | 8 (73) | 0 (0) | 1 (9) | 0 (0) |
| 49n) Lymphadenopathy | 1 (9) | 2 (18) | 5 (45) | 3 (27) | 0 (0) |
| 49o) Location of adenopathy | 1 (9) | 2 (18) | 5 (45) | 3 (27) | 0 (0) |
| 49p) Bleeding | 6 (55) | 5 (45) | 0 (0) | 0 (0) | 0 (0) |
| 49q) Location of bleeding | 3 (27) | 7 (64) | 1 (9) | 0 (0) | 0 (0) |
| 49r) Level of consciousness (alert, confusion, voice, pain, unresponsive) | 6 (55) | 4 (36) | 1 (9) | 0 (0) | 0 (0) |
| 49s) Seizure | 5 (45) | 4 (36) | 2 (18) | 0 (0) | 0 (0) |
| 49t) Breathing difficulty | 3 (27) | 8 (73) | 0 (0) | 0 (0) | 0 (0) |
| 49u) Shock | 5 (45) | 5 (45) | 1 (9) | 0 (0) | 0 (0) |
| 49v) Renal failure | 7 (64) | 4 (36) | 0 (0) | 0 (0) | 0 (0) |
| 49w) Encephalopathy | 6 (55) | 5 (45) | 0 (0) | 0 (0) | 0 (0) |
| 49x) Respiratory failure | 3 (27) | 8 (73) | 0 (0) | 0 (0) | 0 (0) |
| Assessment of vital signs at post-baseline timepoints | 6 (67) | 3 (33) | 0 (0) | 0 (0) | 0 (0) |
| 50a) Respiratory rate | 6 (55) | 5 (45) | 0 (0) | 0 (0) | 0 (0) |
| 50b) Pulse | 7 (64) | 4 (36) | 0 (0) | 0 (0) | 0 (0) |
| 50c) Blood pressure | 7 (64) | 4 (36) | 0 (0) | 0 (0) | 0 (0) |
| 50d) Temperature | 9 (82) | 2 (18) | 0 (0) | 0 (0) | 0 (0) |
| 50e) Oxygen saturation | 6 (55) | 4 (36) | 1 | 0 (0) | 0 (0) |
| 50f) Cardiac function (ECG) | 1 (9) | 7 (64) | 3 (27) | 0 (0) | 0 (0) |
| 50g) AST | 3 (27) | 7 (64) | 1 (9) | 0 (0) | 0 (0) |
| 50h) ALT | 3 (27) | 7 (64) | 1 (9) | 0 (0) | 0 (0) |
| 50i) Creatinine | 6 (55) | 5 (45) | 0 (0) | 0 (0) | 0 (0) |
| 50j) BUN | 4 (36) | 7 (64) | 0 (0) | 0 (0) | 0 (0) |
| 50k) Urine protein | 4 (36) | 5 (45) | 1 (9) | 1 (9) | 0 (0) |
| Outcome at discharge | 11 (100) | 0 (0) | 0 (0) | 0 (0) | 0 (0) |
| Outcome upon completion of all trial visits | 7 (64) | 2 (18) | 1 (9) | 1 (9) | 0 (0) |
| Date of outcome | 8 (73) | 3 (27) | 0 (0) | 0 (0) | 0 (0) |

**Table E – Additional items for consideration proposed by stakeholders in Round 1**

| ITEM PROPOSED BY STAKEHOLDERS | N (%) |
| --- | --- |
| **Core Eligibility Criteria: Inclusion criteria** |  |
| Facial swelling | 2 (17%) |
| Hearing loss | 2 (17%) |
| Renal failure | 1 (8%) |
| Jaundice | 1 (8%) |
| Breast engorgement | 1 (8%) |
| Conjunctivitis | 1 (8%) |
| **Core Eligibility Criteria: Exclusion criteria** |  |
| Moribund patients | 1 (8%) |
| Neutrophilia | 1 (8%) |
| Bleeding without associated fever | 1 (8%) |
| Patients on end-of-life care | 1 (8%) |
| Patients involved in another clinical trial | 1 (8%) |
| **Core Outcome Set: Outcome measures** |  |
| Survival with complications | 1 (8%) |
| Haemoglobin level and other haematologic parameters | 1 (8%) |
| Renal function | 1 (8%) |
| Malignancies | 1 (8%) |
| Resolution of fever | 1 (8%) |
| Time to hospital discharge | 2 (17%) |
| Time to PCR negative result | 1 (8%) |
| Time to defervescence | 1 (8%) |
| Presence of anaemia | 1 (8%) |
| Hearing loss | 1 (8%) |
| Outcome of subsequent pregnancies | 1 (8%) |
| Vertical transmission | 1 (8%) |
| Time to delivery/ miscarriage | 1 (8%) |
| Time between treatment start and delivery/miscarriage | 1 (8%) |
| **Core Data Variables** |  |
| Bleeding assessed as micro- and frank bleeds | 1 (8%) |
| Edema/ swelling | 2 (17%) |
| Pharyngitis | 2 (17%) |
| Retrosternal pain | 1 (8%) |
| Liver failure | 1 (8%) |
| Jaundice | 1 (8%) |
| Hepatitis | 1 (8%) |
| Renal insufficiency/ oliguria | 1 (8%) |
| White blood cell count | 2 (17%) |
| White blood cell differential | 2 (17%) |
| Full blood count | 2 (17%) |
| Total body protein | 2 (17%) |
| Haemoglobin | 2 (17%) |
| Leukocytes | 1 (8%) |
| Malaria diagnostics | 1 (8%) |
| Sodium | 1 (8%) |
| Potassium | 1 (8%) |
| Urinalysis | 1 (8%) |
| Point of care ultrasound (pregnant women) | 1 (8%) |
| Vaginal bleeding (pregnant women) | 1 (8%) |

**Table F – points for further discussion as proposed by stakeholders**

| POINTS FOR FURTHER DISCUSSION |  |
| --- | --- |
| **Core Eligibility Criteria** | How to incorporate asymptomatic or mild cases in to a trial’s eligibility criteria |
| **Core Case Definition** | Acceptability of using RT-PCR alone to diagnose LF |
|  | How to perform laboratory case confirmation where RT-PCR is not available |
|  | How to identify a confirmed case where it is not possible to conduct a laboratory diagnosis |
| **Core Outcome Measures** | Development of acceptable definitions of clinical syndromes included as outcome measures |

***Results from Round 2***

**Table G – Frequency and mean of responses to items proposed for inclusion in the Core Eligibility Criteria**

| ELIGIBILITY CRITERIA | N (%) | | | | |
| --- | --- | --- | --- | --- | --- |
|  | Strongly Agree | Agree | No preference | Disagree | Strongly disagree |
| INCLUSION CRITERIA - GENERAL |  |  |  |  |  |
| In order to be included in a LF clinical trial, patients need to have a clinical diagnosis of LF AND positive result on RT-PCR for LF. | 17 (74) | 6 (26) | 0 (0) | 0 (0) | 0 (0) |
| FOR INVESTIGATIONAL DRUGS WITH A LOW-RISK SAFETY PROFILE |  |  |  |  |  |
| In the absence of RT-PCR or delays to receiving the RT-PCR result, it would be acceptable for patients to be included in a clinical trial on the basis of clinical diagnosis alone | 5 (21) | 3 (13) | 3 (13) | 10 (42) | 3 (13) |
| FOR INVESTIGATIONAL DRUGS WITH A HIGH-RISK SAFETY PROFILE |  |  |  |  |  |
| In order to be included in a LF clinical trial, patients need to have a clinical diagnosis of LF AND positive result on RT-PCR for LF. In the absence of RT-PCR or delays to receiving the RT-PCR result, patients should not be included | 12 (52) | 10 (43) | 1 (4) | 0 (0) | 0 (0) |
| DEFINITION OF "CLINICAL DIAGNOSIS" |  |  |  |  |  |
| Clinical diagnosis is defined as the presence of fever that has not responded to treatment, plus ONE OR MORE pre-specified signs and symptoms (**see Table S8**) | 9 (41) | 9 (41) | 2 (9) | 2 (9) | 0 (0) |
| PAEDIATRIC POPULATION |  |  |  |  |  |
| Subjective signs and symptoms, like headache, should be removed from the pre-defined list for children under 5 years old | 8 (35) | 9 (40) | 6 (26) | 0 (0) | 0 (0) |
| EXCLUSION CRITERIA |  |  |  |  |  |
| Patients with malaria should be excluded from Lassa fever clinical trials | 0 (0) | 1 (4) | 0 (0) | 18 (75) | 5 (21) |
| Patients with bacterial co-infection should be excluded from Lassa fever clinical trials | 0 (0) | 6 (25) | 2 (8) | 13 (54) | 3 (13) |
| Patients with acute-infections leading to fever should be excluded from Lassa fever clinical trials | 1 (4) | 5 (21) | 7 (29) | 10 (41) | 1 (4) |
| Patients with neutrophilia should be excluded from Lassa fever clinical trials | 0 (0) | 3 (13) | 11 (45) | 7 (29) | 3 (13) |
| Patients with bleeding without associated fever should be excluded from Lassa fever clinical trials | 2 (8) | 5 (21) | 3 (13) | 11 (45) | 3 (13) |
| Moribund patients should be excluded from Lassa fever clinical trials | 5 (21) | 9 (38) | 3 (13) | 6 (25) | 1 (4) |
| Patients on end-of-life care should be excluded from Lassa fever clinical trials | 5 (21) | 12 (50) | 1 (4) | 6 (25) | 0 (0) |
| Patients involved in another clinical trial should be excluded from Lassa fever clinical trials | 7 (29) | 13 (54) | 2 (8) | 2 (8) | 0 (0) |

**Table H – Frequency of responses to items proposed for inclusion as signs and symptoms that should be present for clinical diagnosis of LF**

| SIGN/SYMPTOM | Fundamental to LF diagnosis | Not usually indicative of LF |
| --- | --- | --- |
| Fever | 21 (91) | 2 (9) |
| Back or joint pain | 4 (17) | 19 (83) |
| Weakness | 11 (48) | 12 (52) |
| Headache | 7 (30) | 16 (70) |
| Chest pain | 7 (30) | 16 (70) |
| Malaise | 11 (48) | 12 (52) |
| Sore throat | 16 (70) | 7 (30) |
| Abdominal pain | 9 (39) | 14 (41) |
| Vomiting | 7 (30) | 16 (70) |
| Nausea | 7 (30) | 16 (70) |
| Diarrhoea | 7 (30) | 16 (70) |
| Bleeding from orifices | 20 (87) | 3 (13) |
| Generalised muscle pain | 10 (43) | 13 (57) |
| Facial swelling | 9 (39) | 14 (61) |
| Hearing loss | 14 (61) | 9 (39) |
| Renal failure | 9 (41) | 13 (59) |
| Jaundice | 4 (17) | 19 (83) |
| Breast engorgement | 10 (43) | 13 (57) |
| Neck pain | 0 (0) | 23 (100) |
| Conjunctivitis | 9 (39) | 14 (61) |

**Table I – Frequency and mean of responses to items proposed for inclusion in the Core Case Definition**

| CASE DEFINITION | N (%) | | | | |
| --- | --- | --- | --- | --- | --- |
|  | Strongly agree | Agree | No preference | Disagree | Strongly disagree |
| CONFIRMED CASE DEFINTION |  |  |  |  |  |
| A confirmed case is defined as a patient who has ONE OR MORE signs or symptoms of Lassa fever AND who has a confirmed laboratory diagnosis | 19 (79) | 5 (21) | 0 (0) | 0 (0) | 0 (0) |
| SITUATIONS WHERE IT IS NOT POSSIBLE TO PERFORM LABORATORY DIAGNOSIS |  |  |  |  |  |
| Locations where it is not possible to perform laboratory diagnosis of Lassa fever should **not** be able to participate in a clinical trial | 9 (38) | 9 (38) | 0 (0) | 6 (25) | 0 (0) |
| Locations where it is not possible to perform laboratory diagnosis of Lassa fever should be able to participate in a clinical trial | 2 (8) | 7 (29) | 0 (0) | 8 (33) | 7 (29) |
| **IF YOU 'STRONGLY AGREE' OR 'AGREE' WITH THE ABOVE STATEMENT:** Locations where it is not possible to perform laboratory diagnosis of Lassa fever should consider a confirmed case as a patient who has ONE OR MORE signs or symptoms of Lassa Fever AND who has a confirmed epidemiological link to one or more of the following exposures within three weeks of presentation: 1. Contact with blood or other bodily fluids of a paitent with Lassa fever 2. Residence in or travel to a Lassa fever endemic area 3. Work in a laboratory that handles Lassa fever specimens or that handles rodents from endemic areas 4. Exposure to semen from a confirmed acute or convalescent case of Lassa fever within 10 weeks of that person's onset of symptoms (Definition above taken from 2011 CDC case definition: https://wwwn.cdc.gov/nndss/conditions/lassa-virus/case-definition/2011/) | 5 (45) | 3 (27) | 0 (0) | 2 (18) | 1 (9) |
| CASE CONFIRMATION |  |  |  |  |  |
| RT-PCR alone is sufficient to confirm Lassa fever in a clinical trial | 12 (55) | 10 (45) | 0 (0) | 0 (0) | 0 (0) |
| If RT-PCR is not available, testing with ONE of the following methods is sufficient: 1. Viral culture 2. Immunofluorescence Assay (IFA) 3. ELISA IgM 4. ELISA Ag 5. ELISA IgG 6. Complement Fixation (CF) 7. Immunohistochemistry (IHC) | 4 (17) | 8 (34) | 1 (4) | 7 (30) | 3 (13) |
| If RT-PCR is not available, testing using TWO OR MORE methods (as below) should be acceptable for case confirmation: 1. Viral culture 2. Immunofluorescence Assay (IFA) 3. ELISA IgM 4. ELISA Ag 5. ELISA IgG 6. Complement Fixation (CF) 7. Immunohistochemistry (IHC) | 5 (24) | 10 (47) | 1 (5) | 4 (19) | 1 (5) |

**Table SJ – Frequency of selections of acceptable laboratory methods of case confirmation**

| CASE DEFINITION | N (%) | | | | | | |
| --- | --- | --- | --- | --- | --- | --- | --- |
|  | Viral culture | IFA^1^ | ELISA IgM | ELISA Ag | ELISA IgG | CF^2^ | IHC^3^ |
| **IF YOU 'STRONGLY AGREE' OR 'AGREE' WITH ITEM 41:** Please state ONE method that you would find acceptable to perform ALONE to confirm Lassa fever in the absence of RT-PCR | 6 (46) | 1 (8) | 4 (31) | 2 (15) | 0 (0) | 0 (0) | 0 (0) |

^1^Immunofluorescence Assay; ^2^ Complement Fixation; ^3^ Immunohistochemistry

**Table K – Frequency of selections of acceptable laboratory methods of case confirmation**

| CASE CONFIRMATION | N (%) | | | | | | |
| --- | --- | --- | --- | --- | --- | --- | --- |
|  | Viral culture + IFA^1^ | Viral culture + ELISA IgM | IFA^1^ + ELISA Ag | ELISA IgM + ELISA IgG | Viral culture + ELISA IgG | Viral culture + ELISA Ag | ELISA IgM + ELISA IgG + ELISA Ag |
| **IF YOU 'STRONGLY AGREE' OR 'AGREE' WITH ITEM 43:** Please state your preferred COMBINATION of methods that you would find acceptable for case confirmation (STATE ONLY ONE COMBINATION FROM OPTIONS 1-7 BELOW): 1. Viral culture 2. Immunofluorescence Assay (IFA) 3. ELISA IgM 4. ELISA Ag 5. ELISA IgG 6. Complement Fixation (CF) 7. Immunohistochemistry (IHC) | 2 (15) | 4 (31) | 2 (15) | 2 (15) | 1 (8) | 1 (8) | 1 (8) |

^1^Immunofluorescence Assay

**Table L – Frequency of responses to the outcome measures proposed for inclusion in the Core Outcome Set**

| CORE OUTCOME SET | N (%) | | | N (%) | | | |
| --- | --- | --- | --- | --- | --- | --- | --- |
|  | Primary outcome measure | Secondary outcome measure | Not an outcome measure | 14 days | 21 days | 28 days | More than 28 days |
| OUTCOME MEASURES - from the list below, please state whether the outcome measures should be considered 'primary outcome measures', 'secondary outcome measures' or 'not an outcome measure'. For each primary outcome measure, please state the timepoint that it should be assessed. | | | | | | | |
| Survival/ mortality | 22 (96) | 1 (4) | 0 (0) | 10 (59) | 2 (12) | 3 (18) | 2 (12) |
| Survival with complications | 8 (35) | 13 (57) | 2 (9) | 1 (7) | 1 (7) | 5 (36) | 7 (50) |
| Haemoglobin level and other haematologic parameters | 5 (22) | 12 (52) | 6 (26) | 7 (58) | 2 (17) | 3 (25) | 0 (0) |
| Renal function | 9 (39) | 13 (57) | 1 (4) | 5 (38) | 3 (23) | 5 (38) | 0 (0) |
| Resolution of fever | 10 (43) | 6 (26) | 7 (30) | 10 (100) | 0 (0) | 0 (0) | 0 (0) |
| Malignancies | 0 (0) | 3 (14) | 19 (86) | 0 (0) | 0 (0) | 0 (0) | 2 (100) |
| Progression to severe disease (as defined by presence of either renal failure, encephalopathy, shock, respiratory failure) | 15 (65) | 8 (35) | 0 (0) | 9 (60) | 1 (7) | 4 (29) | 0 (0) |
| Unfavourable outcome' (mortality + progression to severe disease) | 18 (78) | 5 (22) | 0 (0) | 9 (60) | 3 (20) | 2 (13) | 1 (7) |
| Time to hospital discharge | 8 (35) | 13 (57) | 2 (9) | 5 (42) | 4 (33) | 2 (17) | 1 (8) |
| Time to PCR negativity | 11 (48) | 11 (48) | 1 (4) | 8 (57) | 1 (7) | 4 (29) | 1 (7) |
| Time to cessation of fever | 9 (39) | 8 (35) | 6 (26) | 9 (82) | 0 (0) | 1 (9) | 1 (9) |
| Time to cessation of symptoms | 8 (35) | 9 (39) | 6 (26) | 8 (73) | 0 (0) | 2 (18) | 1 (9) |
| Presence of severe anaemia | 3 (13) | 16 (70) | 4 (17) | 8 (67) | 2 (17) | 1 (8) | 1 (8) |
| Hearing loss | 6 (26) | 15 (65) | 2 (9) | 2 (17) | 3 (25) | 2 (17) | 5 (42) |
| AST | 4 (18) | 13 (59) | 5 (23) | 7 (58) | 4 (33) | 1 (8) | 0 (0) |
| ALT | 4 (18) | 12 (55) | 6 (27) | 6 (55) | 3 (27) | 2 (18) | 0 (0) |
| Outcome of subsequent pregnancies | 0 (0) | 7 (32) | 15 (68) | 1 (20) | 0 (0) | 0 (0) | 4 (80) |
| Vertical transmission **(PREGNANT PATIENTS ONLY)** | 8 (36) | 11 (50) | 3 (14) | 4 (42) | 2 (17) | 2 (17) | 5 (25) |
| Time between delivery/miscarriage and hospital discharge/recovery **(PREGNANT PATIENTS ONLY)** | 4 (18) | 15 (68) | 3 (14) | 5 (42) | 2 (17) | 2 (17) | 3 (25) |
| Time between treatment start and delivery/miscarriage **(PREGNANT PATIENTS ONLY)** | 4 (18) | 16 (73) | 2 (9) | 4 (33) | 2 (17) | 0 (0) | 6 (50) |
| Pregnancy outcome **(PREGNANT PATIENTS ONLY)** | 13 (62) | 8 (38) | 0 (0) | 3 (27) | 2 (18) | 1 (9) | 5 (45) |

**Table M - Frequency of responses for the selection of a preferred primary outcome measure**

| PREFERRED PRIMARY OUTCOME MEASURE | N (%) |
| --- | --- |
| Survival/Mortality | 13 (59) |
| Unfavourable outcome' (mortality + progression to severe disease) | 3 (14) |
| Maternal and perinatal survival | 2 (9) |
| Discharge without complications | 1 (5) |
| Progression to severe disease/No progression to severe disease | 1 (5) |
| Time to negative result | 1 (5) |
| Vertical transmission | 1 (5) |

**Table N - Frequency of responses for the delineation of the definition for Acute Kidney Injury**

| PROPOSED DEFINITION | N (%) stakeholders who agree with definition | N (%) of stakeholders who disagree with definition |
| --- | --- | --- |
| A rise in serum creatinine of 26 mmol/L or greater within 48 hours | 19 (90) | 2 (10) |
| A 50% or greater rise in serum creatinine known or presumed to have occurred within the past 7 days | 18 (82) | 4 (18) |
| A fall in urine output to less than 0.5 ml/kg/hour for more than 6 hours in adults and more than 8 hours in children and young people | 20 (91) | 2 (9) |
| Decision of the treating physician to start dialysis | 13 (59) | 9 (41) |

**Table O - Frequency of responses for the delineation of the definition for respiratory failure**

| PROPOSED DEFINITION | N (%) stakeholders who agree with definition | N (%) of stakeholders who disagree with definition |
| --- | --- | --- |
| Pulse oximetry: SpO2 <90% | 19 (86) | 3 (14) |
| Pulse oximetry: SpO2 <90% AND decision to start O2 therapy | 17 (77) | 5 (23) |

**Table P - Frequency of responses for the delineation of the definition for shock**

| PROPOSED DEFINITION | N (%) | | | | |
| --- | --- | --- | --- | --- | --- |
|  | Strongly agree | Agree | No preference | Disagree | Strongly disagree |
| The definition of shock should be based on  MAP < 65mmHg + lactate > 2 mmol/L | 4 (19) | 14 (67) | 0 (0) | 3 (14) | 0 (0) |

**Table Q – Frequency of responses for inclusion of encephalopathy as a pathology indicating progression to severe disease**

| PROPOSED DEFINITION | N (%) stakeholders who agree that encephalopathy should be included | N (%) stakeholders who agree that encephalopathy should be excluded |
| --- | --- | --- |
| Should encephalopathy be included as a syndrome indicating 'Progression to severe disease' | 16 (73) | 6 (27) |

**Table R – Additional proposals for the definitions of the pathologies indicating progression to severe disease**

| SYNDROME | PROPOSED DEFINITIONS BY STAKEHOLDERS |
| --- | --- |
| Acute Kidney Injury | KDIGO classification |
|  | SOFA score |
|  | Increase in serum creatinine to 2.0 to 2.9 times baseline, or reduction in urine output to <0.5 mL/kg/hour for ≥12 hours |
|  | Anuria |
| Respiratory failure | SPO2 < 90 on room air, RR > 20/min or < 10/min, use of accessory muscles +/- nasal flaring |
|  | Tachypnoea |
|  | Definition based on clinical signs |
|  | SOFA score |
|  | Absence of respiratory efforts |
| Shock | Any patient with systolic BP less than 100mmHg, diastolic less than 60mmHg, with tachycardia more than 100bpm |
|  | PR > 90/min, decreased capillary refill, decreased urine output, RR > 20/min, systolic < 90, +/- altered level of consciousness, +/- temp > 38 or < 36 |
|  | Clinical definition: obtundation, Heart rate > 100, Respiratory rate > 22, Hypotension (systolic blood pressure < 90 mm Hg) or a 30-mm Hg fall in baseline blood pressure), Urine output < 0.5 mL/kg/hour |
|  | Systolic BP <90mmhg |
|  | qSOFA: Tachycardia, tachypnoea, low BP, oliguria |
|  | High (>120/min), thready pulse rate or impalpable pulse with extremely low systolic (< 60 mmHg) and diastolic (< 40 mmHg) blood pressure (measured on the arm). |
|  | Use of bicarbonate levels lower than 15mmol/L can be used as an indication of acidosis |
|  | SOFA score |
|  | Hypotension, cold and clammy extremities |
| Encephalopathy | Any patient presenting with altered sensorium that cannot be explained by hypertension, uraemia, liver failure and head injury etc. |
|  | Encephalopathy refers to a clinical state of altered mental status, manifesting as confusion, disorientation, behavioural changes or other cognitive impairments with or without inflammation of brain tissue.  Https://doi.org\|10.1016\|8978-0-323-53088-0.00001-4 |
|  | Altered mental function not explained by measurable biochemical derangements e.g. blood sugar, liver function, renal function or respiratory functional parameters |
|  | Presence of alteration of consciousness (GCS score <12/15) and/or seizures |
|  | Deteriorating Glasgow coma score or impaired alertness/consciousness |
|  | Using the ACVPU score which is simple and reproducible (A = alert, C=confusion, V=verbal, P=pain, U=unresponsive) |
|  | Abnormal hypo- and/or hyper-manifestations of brain functions seen in or in association with a disease condition. |
|  | Unexplained deterioration in brain function - evidenced by presence of one or more of the following: seizures, confusion/irrational behaviours/talk, unconsciousness, cranial nerve deficits, hyper- or hypotonia/reflexia, NOT EXPLAINED by other comorbidities like presence of bacterial meningitis |
|  | SOFA |
|  | List of encephalopathy signs/symptoms: seizure, coma, delir, confusion in absence of electrolyte disturbances or other aetiologies |
|  | Presence of disorientation/confusion or loss of consciousness |

**Table S - Frequency of responses for the items proposed for inclusion in the Core Data Set**

| CORE DATA VARIABLES | N (%) | | |
| --- | --- | --- | --- |
|  | Critical to evaluate treatment efficacy and/or safety | Important but not critical to evaluate treatment efficacy and/or safety | Not important |
| Fever | 22 (92) | 1 (4) | 1 (4) |
| Weakness | 6 (25) | 15 (63) | 3 (13) |
| Dizziness | 3 (13) | 12 (50) | 9 (38) |
| Muscle pain | 3 (13) | 16 (67) | 5 (21) |
| Chest pain | 4 (17) | 15 (63) | 5 (21) |
| Back pain | 2 (8) | 16 (67) | 6 (25) |
| Joint pain | 1 (4) | 16 (67) | 7 (29) |
| Malaise | 5 (21) | 17 (71) | 2 (8) |
| Sore throat | 10 (42) | 12 (50) | 2 (8) |
| Abdominal pain | 4 (17) | 17 (71) | 3 (13) |
| Vomiting | 4 (17) | 15 (65) | 4 (17) |
| Nausea | 1 (4) | 16 (67) | 7 (29) |
| Diarrhoea | 6 (25) | 13 (54) | 5 (21) |
| Bleeding (general) | 21 (88) | 3 (13) | 0 (0) |
| Frank bleeding | 20 (83) | 4 (17) | 0 (0) |
| Micro bleeding | 13 (54) | 11 (46) | 0 (0) |
| Level of consciousness | 21 (88) | 3 (13) | 0 (0) |
| Seizure | 14 58) | 10 (42) | 0 (0) |
| Breathing difficulty | 13 (54) | 10 (42) | 1 (4) |
| Labour complications | 13 (54) | 9 (38) | 2 (8) |
| Breast tenderness | 4 (17) | 14 (58) | 6 (25) |
| Edema or swelling | 9 (38) | 13 (54) | 2 (8) |
| Pharyngitis | 8 (33) | 12 (50) | 4 (17) |
| Retrosternal pain | 4 (17) | 16 (67) | 4 (17) |
| Liver pain | 3 (13) | 13 (54) | 8 (33) |
| Jaundice | 7 (29) | 11 (46) | 6 (25) |
| Hepatitis | 9 (38) | 9 (38) | 6 (25) |
| Renal insufficiency | 21 (88) | 3 (13) | 0 (0) |
| Oliguria | 20 (83) | 4 (17) | 0 (0) |
| Pregnancy complications | 20 (87) | 3 (13) | 0 (0) |
| Vaginal bleeding | 15 (65) | 8 (35) | 0 (0) |
| Excessive bleeding during labour | 14 (61) | 9 (39) | 0 (0) |
| Respiratory rate | 16 (67) | 7 (29) | 1 (4) |
| Pulse | 18 (75) | 6 (25) | 0 (0) |
| Blood pressure | 21 (88) | 3 (13) | 0 (0) |
| Temperature | 23 (96) | 1 (4) | 0 (0) |
| Oxygen saturation | 18 (75) | 6 (25) | 0 (0) |
| Cardiac function | 10 (42) | 14 (58) | 0 (0) |
| AST | 20 (83) | 3 (13) | 1 (4) |
| ALT | 18 (75) | 5 (21) | 1 (4) |
| Creatinine | 24 (100) | 0 (0) | 0 (0) |
| BUN | 21 (88) | 3 (13) | 0 (0) |
| Urine protein | 9 (38) | 15 (63) | 0 (0) |
| White blood cell and differential | 13 (54) | 11 (46) | 0 (0) |
| Full blood count | 12 (50) | 12 (50) | 0 (0) |
| Total body protein | 7 (29) | 13 (54) | 4 (17) |
| Albumin | 10 (42) | 11 (46) | 3 (13) |
| Globulin | 7 (29) | 13 (54) | 4 (17) |
| Haemoglobin | 16 (70) | 7 (30) | 0 (0) |
| Leukocytes | 11 (48) | 11 (48) | 1 (4) |
| Sodium | 10 (42) | 11 (46) | 3 (13) |
| Potassium | 17 (71) | 7 (29) | 0 (0) |
| Urinalysis | 11 (46) | 13 (54) | 0 (0) |
| Point of care ultrasound **(PREGNANT PATIENTS ONLY)** | 14 (61) | 9 (39) | 0 (0) |

**Table T - Frequency of responses for other considerations in the Core Data Set**

| ITEM | N (%) |  |  |  |  |
| --- | --- | --- | --- | --- | --- |
|  | Strongly agree | Agree | No preference | Disagree | Strongly disagree |
| The date of contact with a confirmed case should be optional | 3 (13) | 13 (54) | 0 (0) | 6 (25) | 2 (8) |
| Collection of data relating to lymphadenopathy to be removed as a core data variable | 6 (25) | 12 (50) | 3 (13) | 3 (13) | 0 (0) |
| Collection of data relating to location of adenopathy to be removed as a core data variable | 5 (23) | 10 (45) | 3 (14) | 4 (18) | 0 (0) |
| Bleeding should be assessed in terms of both micro and frank bleeds | 9 (39) | 11 (48) | 2 (9) | 1 (4) | 0 (0) |
| Severity grading should be performed only for Lassa fever complications | 3 (13) | 11 (46) | 1 (4) | 9 (38) | 0 (0) |
| Severity grading should not be performed at all and symptoms and syndromes should only be marked as 'present' or 'not present' | 2 (8) | 4 (17) | 2 (8) | 11 (48) | 5 (23) |

***Results of Round 3***

**Table U - Frequency of responses for the definition of clinical diagnosis (Poll 1)**

| PROPOSED DEFINITION | N (%) |
| --- | --- |
| No restrictions on signs and symptoms in order for clinical diagnosis to be made | 6 (23) |
| Presence of fever AND [(at least one of the following: headache/back pain/joint pain) OR (at least one digestive symptom: abdominal pain, vomiting, diarrhoea)] | 1 (4) |
| Presence of fever AND [at least one of the following: headache/weakness/back pain/ joint pain/ dizziness/sore throat/bleeding) OR (at least one digestive symptom: abdominal pain, vomiting, diarrhoea)] | 12 (46) |
| Presence of fever AND at least one of the following: sore throat/bleeding from orifices/ hearing loss | 2 (8) |
| In the absence of fever, presence of [(at least one of the following: headache/backache/joint pain) AND (at least one digestive symptom: abdominal pain, vomiting, diarrhoea)] | 5 (19) |

**Table V - Frequency of responses for the definition of clinical diagnosis (Poll 2)**

| PROPOSED DEFINITION | N (%) |
| --- | --- |
| No restrictions on signs and symptoms in order for clinical diagnosis to be made | 2 (9) |
| History of fever or presence of fever unresponsive to treatment for common illnesses AND [at least one of the following: headache/weakness/back pain/ joint pain/ dizziness/sore throat/bleeding) OR (at least one digestive symptom: abdominal pain, vomiting, diarrhoea)] – expanded to include signs and symptoms associated with Lassa fever in pregnancy (vaginal bleeding; abortion or miscarriage; unexplained intrauterine death; unexplained breast engorgement) | 14 (64) |
| History of fever or presence of fever unresponsive to treatment for common illnesses, presence of [(at least one of the following: headache/weakness/back pain/ joint pain/ dizziness/sore throat/bleeding) AND (at least one digestive symptom: abdominal pain, vomiting, diarrhoea)] - expanded to include signs and symptoms associated with Lassa fever in pregnancy (vaginal bleeding; abortion or miscarriage; unexplained intrauterine death; unexplained breast engorgement) | 6 (27) |

**Table W – Frequency of responses for selecting ‘unfavourable outcome’ as the primary outcome measure**

| PRIMARY OUTCOME MEASURE | N (%) |
| --- | --- |
| Agree | 19 (83) |
| Disagree | 4 (17) |

**Table X – Frequency of responses for assessing the primary outcome measure at 14 days**

| PRIMARY ENDPOINT | N (%) |
| --- | --- |
| Agree | 21 (84) |
| Disagree | 4 (16) |

**Table Y – Frequency of responses for defining the assessment of Acute Kidney Injury (AKI)**

| DEFINITION OF ACUTE KIDNEY INJURY | N (%) |
| --- | --- |
| SOFA 0-4 (creatinine or urine output) | 8 (36) |
| SOFA 0-4 OR urine output alone are acceptable | 14 (64) |
| Urine output alone | 0 (0) |

**Table Z – Frequency of responses for defining the assessment of Acute Respiratory Distress Syndrome (ARDS)**

| DEFINITION OF ACUTE RESPIRATORY DISTRESS SYNDROME | N (%) |
| --- | --- |
| SOFA 0-4 (PaO_2_/FiO_2_) | 1 (5) |
| SOFA 0-4 (PaO2/FiO2) OR SOFA 0-4 (SF [SPO2/FIO2]) are acceptable | 15 (68) |
| SOFA 0-4 (SF [SPO_2_/FIO_2_]) | 6 (27) |

**Table AA – Frequency of responses for defining the assessment of shock**

| DEFINITION OF SHOCK | N (%) |
| --- | --- |
| SOFA 0-4 | 1 (5) |
| SOFA 0-4 OR SOFA 0-4 (with option to not record inotropes if unavailable) are acceptable | 16 (76) |
| SOFA 0-4 (with option to not record inotropes if unavailable) | 4 (19) |

**Table AB – Frequency of responses for defining the assessment of encephalopathy (Poll 1)**

| DEFINITION OF ENCEPHALOPATHY | N (%) |
| --- | --- |
| SOFA 0-4 (Glasgow Coma Scale) | 3 (16) |
| SOFA 0-4 (Glasgow Coma Scale) OR AVPU score | 15 (79) |
| AVPU score | 1 (5) |

**Table AC – Frequency of responses for defining the assessment of encephalopathy (Poll 2)**

| DEFINITION OF ENCEPHALOPATHY | N (%) |
| --- | --- |
| ACVPU | 24 (100) |
| Glasgow Coma Scale | 0 (0) |

**Table AD – Frequency of responses for defining the assessment of encephalopathy (Poll 3)**

| DEFINITION OF ENCEPHALOPATHY | N (%) |
| --- | --- |
| ACVPU + seizure | 15 (88) |
| ACVPU alone | 2 (12) |
